# Scientific Impact on Socially Beneficial Behaviors: Impact and Efficiency Evidence From Behavior Change Interventions

**DOI:** 10.1111/spc3.70109

**Published:** 2025-12-04

**Authors:** Dolores Albarracin

**Affiliations:** Department of Psychology and Annenberg School for Communication, University of Pennsylvania, Philadelphia, Pennsylvania, USA

## Abstract

Psychology and related fields have developed a science of behavior change that supports interventions in various areas, including health, sustainability, and education. This review assesses the contributions of each discipline to the performance (i.e., efficacy) and efficiency (i.e., cost-effectiveness) of these interventions. We analyzed published experimental studies targeting 16 intervention targets, including knowledge, beliefs, social norms, social support, access, and habits. For each target, we estimated its prevalence in the literature, its efficacy in changing behavior, and its cost-effectiveness in improving outcomes. We then used GPT to assess the degree to which 14 academic disciplines (e.g., social psychology, psychology, communication, economics, and engineering) are linked to each target, based on their centrality to conceptualizing and measuring it. These disciplinary ratings were combined with target efficacy and cost-effectiveness to estimate each discipline’s overall contribution to behavioral change. Psychology, including social and cognitive psychology, made the largest contribution to efficacious interventions, followed by communication, public health, and medicine. Economics and neuroscience were associated with the most cost-effective interventions, although all disciplines fell significantly below a common benchmark for cost-effectiveness in the health sector (i.e., $50,000 QALY). Despite its efficacy and cost-effectiveness, psychology receives relatively limited federal funding compared to other disciplines.

## 1 | Introduction

In 1969, George A. Miller delivered his presidential address at the American Psychological Association’s meeting in Washington, D.C. In his call to “give psychology away,” he proposed that psychology could move beyond scientific understanding to promote socially beneficial behavior. Now that more than 50 years have gone by, it is time to ask: How is psychology doing? What are the effects of psychological science on the development of interventions to promote socially beneficial behavior? Is psychological science informing policies, interventions, and technologies that improve human welfare? Is this influence quantifiable, and does it produce any economic benefits? How does psychology compare with other disciplines in terms of measurable effects on behavior, and what is the cost of those effects?

In this paper, we quantify the contribution of psychology, relative to other disciplines (e.g., medicine and economics), to the efficacy and cost-effectiveness of behavior change interventions. In an era of increasing scrutiny of science, psychologists must measure and communicate their social impact with data. To date, the social impact of psychology has been measured through qualitative analyses of cases (Miller 1969) and studies of media dissemination (Evans 2025). For example, in 2025, the American Psychological Association (APA) reported a 1000% increase in media mentions of APA and APA journal publications since 2018 (Evans 2025). Thus, supplementing these efforts, this paper introduces a possible quantification of the impact of psychology, social psychology, and other disciplines by examining their intellectual effect on the performance (i.e., efficacy) and efficiency (i.e., cost-effectiveness) of behavior change interventions in the scientific literature.

Beyond a purely academic exercise, quantifying the scientific impact on behavioral interventions has practical implications for research policy, funding, and cross-disciplinary collaboration. Estimates of efficacy and cost-effectiveness can guide funders in allocating resources toward disciplines and programs with proven societal benefits. Also, understanding which disciplines contribute most to effective interventions highlights where interdisciplinary partnerships can be most productive, ensuring that psychological insights inform engineering, medical, and policy applications. Even if imperfect, attempting quantification is an invitation for more concerted research efforts to track disciplinary impact in systematic ways.

### 1.1 | Behavior Change Interventions

Many contemporary problems require changes to behavior, whether diet, physical activity, or environmentally sustainable actions. Behavior change interventions are structured attempts to change observable actions or inactions, often measured through Randomized Controlled Trials (RCTs). Interventions can be designed to instill new norms, train individuals in new routines, or supply a universal income to influence behavior (e.g., improving diet) and other outcomes (e.g., curbing diabetes). In intervention research terminology, *efficacy* refers to an intervention’s impact as measured by the outcomes of an experiment or randomized controlled trial, such as whether experimental participants have changed their diet more than control participants. *Effectiveness* encompasses broader impacts beyond the context of efficacy research, such as applying an intervention previously tested through an efficacy trial to an entire country. Within effectiveness, *cost-effectiveness* involves the cost of achieving an outcome, such as a year of good health for the average recipient of an intervention. A common benchmark is for health programs to be considered cost-effective when the cost per quality-adjusted life year (QALY) gained is less than $50,000.

Assessing disciplinary contributions to socially beneficial behavior poses several challenges. One is to measure the efficacy and cost-effectiveness of different interventions. Fortunately, intervention efficacy data are plentiful, and some cost-effectiveness data are available. Another challenge is to assess how much a discipline, such as psychology, contributes to defining and measuring variables addressed by the interventions (i.e., *intervention targets*). e.g., one could establish how much a particular concept, such as *attitude*, is connected to psychology or chemistry. Once that link has been made, one could determine how much a discipline informs efficacious and cost-effective interventions by tracking its influence on those targets within the universe of interventions in the literature.

This paper integrates four quantitative markers for a set of 16 intervention targets (e.g., knowledge, behavioral efficacy, cost-effectiveness, and descriptive norms, or behavioral skills) identified in a prior review of the impact of behavior change interventions (Albarracín et al. 2024). For each intervention target, we measured (a) references to it within the behavior change intervention literature and (b) the degree to which 14 disciplines, including psychology and social psychology, as well as “placebo” disciplines such as chemistry, played a central role in conceptualizing and measuring the target. We then merged these data with intervention efficacy and cost-effectiveness data to estimate the contribution of each discipline to the average behavior change study in the literature. If the literature is a reliable gauge of what behavior change interventions achieve in the real world, this process also establishes the relative contribution of different disciplines to real-world outcomes. Furthermore, we considered funding equity by discussing the alignment of our disciplinary contribution estimates with the proportion of federal funding allocated to psychology versus other disciplines.

### 1.2 | The Disciplines Behind Behavior Change Intervention Targets

Social psychology has provided the conceptual and empirical foundations for distinguishing the social-structural and individual factors that inform behavior change interventions. A broad range of social psychological theories has formalized individual mechanisms (Ajzen and Fishbein 1980; Bandura 1991, 1997; Bandura and Wood 1989). A recent model delineates 16 social-structural and individual variables that interventions can manipulate with varying levels of success. These appear in Table 1.

The primary individual building blocks of interventions are knowledge, beliefs, attitudes, emotions, skills, and habits, each representing distinct yet interrelated determinants of behavior regulation (see Table 1). Knowledge and beliefs provide informational and probabilistic appraisals that shape various judgments and expectations, while general and behavioral attitudes reflect evaluations of objects and behaviors (Fishbein and Ajzen 2011; Wyer and Albarracín 2014). Emotions constitute visceral reactions that further influence motivational readiness, whereas general and behavioral skills denote cognitive or motor routines enabling behavioral enactment (Bandura 1997; Fisher and Fisher 1992; Fisher et al. 2006). Habits, as automated action patterns maintained independently of conscious intention (Neal et al. 2006), represent an additional mechanism linking repeated behavior to sustained change.

In parallel, social and structural determinants of behavior include norms, social support, trustworthiness, and access (see Table 1). Social psychologists conceptualized injunctive and descriptive norms (Cialdini 1993, 2003; Cialdini and Goldstein 2004), which describe perceived social approval and behavioral prevalence, respectively. Additional constructs such as institutional trustworthiness (Tyler 2017) and social support (Turner et al. 1983) emphasize the relational and institutional contexts that enable or constrain individual action. These advances highlight that behavior change is not solely a product of individual processes but emerges from the continuous interaction between psychological processes and the broader social and structural environment.

Although social psychology has provided many perspectives on behavior change, other disciplines have also made significant contributions to the factors in Table 1. For example, within clinical psychology, motivational interviewing was developed to motivate behavior change by capitalizing on ambivalence and cognitive dissonance (Hettema et al. 2005). Economics has characterized the influence of choice architectures (Thaler and Sunstein 2008) and incentives (Campbell 2018), engineering has contributed electronic systems to deliver knowledge, monitor behavior, and train behavioral skills (Hekler et al. 2018), and sociology has uncovered how social capital and networks facilitate informational and instrumental support (Dubos 2017). Many of these lessons have been amplified within public health and medicine, in part through the health belief model and ecological approaches to health behavioral change (Janz and Becker 1984; Kerr et al. 2012).

Although various disciplines can lay claim to the concepts in Table 1, we are not aware of any systematic method for making these attributions. In this paper, disciplinary contribution is inferred from the definitional and measurement origins of key intervention constructs. Constructs such as *attitude*, *habit*, or *social support* emerged within a distinct disciplinary tradition that provided the theoretical and methodological infrastructure to study them. Tracing these origins can give an accurate representation of disciplinary contributions, emphasizing epistemic and technical ownership.

The correspondence between intervention targets and disciplinary origins can be established through surveys of domain experts or comprehensive literature reviews that document the theoretical and methodological provenance of each construct. However, in practice, both strategies are limited. Expert surveys rely on subjective recall and disciplinary self-attribution, while manual reviews cannot feasibly cover the whole corpus of cross-disciplinary diffusion that characterizes science. However, recent advances in large language models (LLMs) provide an alternative means to approximate such mappings. Trained on extensive scientific and technical corpora, LLMs encode statistical regularities reflecting disciplinary language. These representations can therefore be used to infer the relative centrality of a discipline in defining and operationalizing behavioral constructs. In this study, GPT-based ratings were used to quantify the conceptual associations between 16 standardized intervention targets (Table 1) and 14 academic disciplines, yielding a scalable estimation of disciplinary centrality grounded in the aggregated scientific literature.

### 1.3 | Overview

This review quantified the contributions of various scientific disciplines to socially beneficial behaviors by examining their influence on targets of behavior change interventions. The central logic is that disciplines shape intervention science by conceptualizing and measuring intervention targets (e.g., attitudes, norms, habits, and social support). By examining these targets as bridges between theory and intervention, we can estimate each discipline’s contribution to the practical impact of behavior change interventions.

**FIGURE 1 |:**
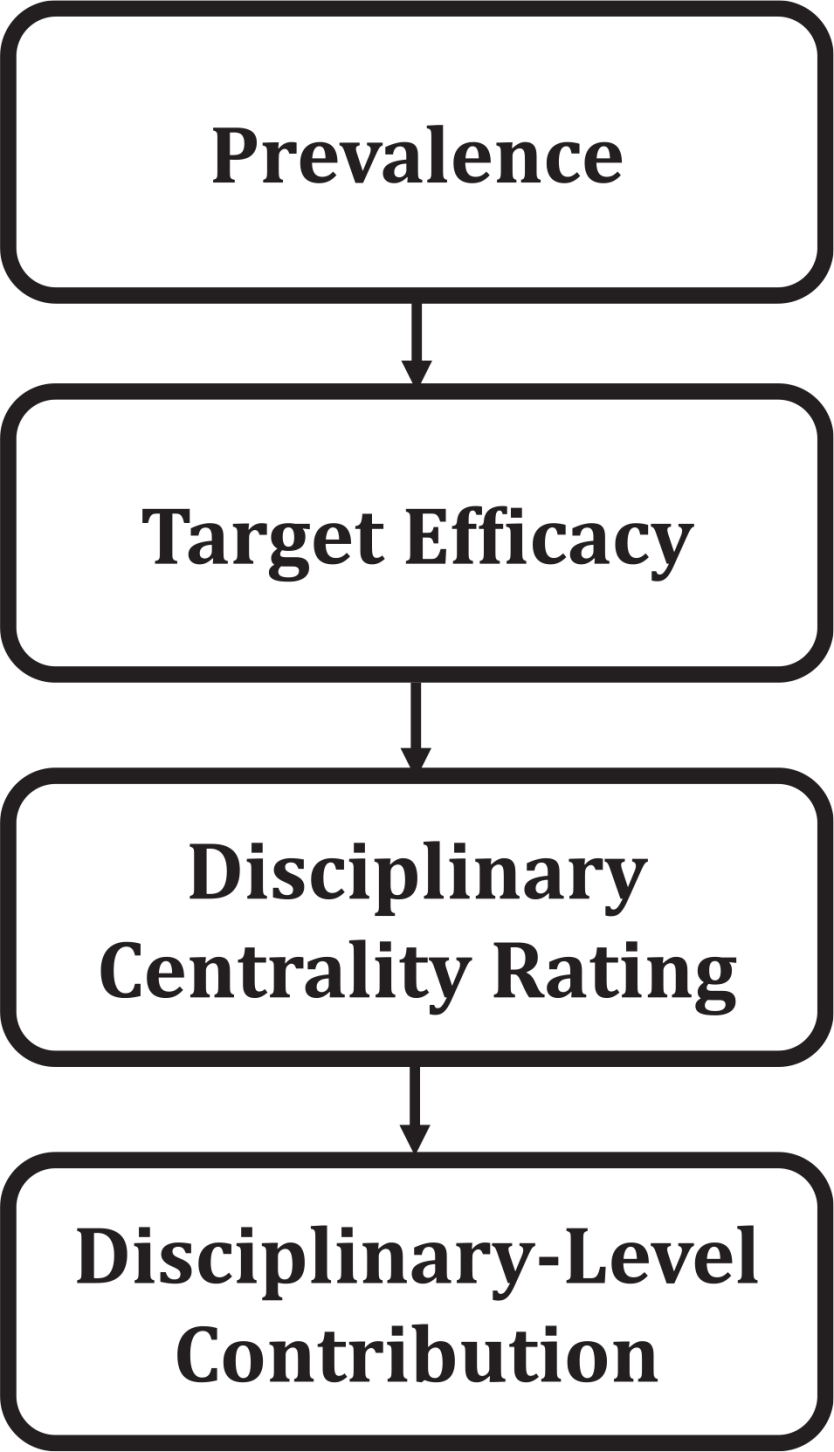
Review process to establish disciplinary contributions to the behavior change intervention literature.

To operationalize this framework, we integrate different quantitative indicators, which are depicted in Figure 1. First, we estimate the prevalence of each intervention target in the experimental behavior-change literature, providing a measure of the target’s representation in the evidence base. Second, we use efficacy and cost-effectiveness data to gauge how much interventions targeting each construct change behavior and at what cost. Third, we create an index of disciplinary centrality, estimating the contributions of each discipline to defining and measuring each target, as derived from GPT-based ratings. Together, these indices yield composite estimates of disciplinary contributions to the efficacy and cost-effectiveness of interventions. In summary, this method combines existing data to evaluate the influence of sciences on practical outcomes.

## 2 | Method

All data and scripts used in this paper can be found here: https://osf.io/3hpsx/overview?view_only=078dda55c4ac45198c9afd28cc3b5652. *R* was used for all analyses (packages: readxl, Dplyr, stringr, tidyr, ggplot2, and readr).

### 2.1 | Sources of Data

This review integrated four sources of information: (a) prevalence of behavior change intervention articles referencing each target listed in Table 1; (b) GPT-5 ratings of the centrality of 14 disciplines in defining and measuring each target; (c) efficacy data by intervention target (Albarracín et al. 2024); and (d) cost-effectiveness by intervention target (Beard et al. 2022).

### 2.2 | Literature Review

#### Keywords.

To conduct our literature searches, we first identified general and target-specific candidate keywords. The general ones were “behavior change”, “intervention,” and “experiment.” The target specific ones were: “knowledge or information,” “belief,” “skill,” “behavioral skill,” “attitude or evaluation,” “attitude toward behavior,” “emotion or mood,” “habit or routine,” “law or regulation or deterrence,” “trust or justice,” “monitor or reminder,” “injunctive norm or subjective norm or personal norm or social approval or moral norm,” “descriptive norm or behavioral norm or role model,” “social support,” “incentive,” and “access or default.” GPT-5 was prompted to suggest additional keywords, which were manually reviewed for conceptual distinctiveness. The final set of keywords appears in the Supporting Information S1.

#### Literature Searches.

On November 6, 2025, a Boolean search was performed on Web of Science using a general query for experimental tests of behavior change interventions. The logic appears in the Supporting Information S1. The records were saved and analyzed with *R* to combine this general search with each of the 16 target-specific keyword sets.

### 2.3 | Target Prevalence

The literature search produced: (a) the total number of intervention articles ($N_{\text{total}}$), and (b) the number of those articles referencing each intervention target $\left(N_{t}\right)$.

#### Target Prevalence.

Target prevalence was defined as the proportion of all intervention articles referencing a given target:

$$P_{t}=\frac{N_{t}}{N_{\text{total}}}.$$

#### Deflation Factor.

Because individual studies often reference multiple targets, we applied a deflation correction to prevent double counting articles. For each target, we computed the proportion of overlapping articles ($p_{\text{overlap}}$), defined as the proportion of articles referencing at least one additional target. The overlap was estimated using R, based on abstracts obtained from Web of Science.

#### Corrected (deflation–adjusted) Prevalence.

Each target’s prevalence was then multiplied by a deflation factor $D_{t}$ that reduces its weight in proportion to its co-occurrence with other targets:

$$D_{t}=0.50+0.50\times \left(1-p_{\text{overlap},t}\right).$$

The corrected (deflation-adjusted) prevalence was then:

$$P_{t}^{*}=\frac{P_{t}\;\times \;D_{t}}{\sum_{j}\left(P_{j}\;\times \;D_{j}\right)}.$$

When targets fully overlap ($p_{\text{overlap}}=1$), $D_{t}=0.5$, halving their contribution. When targets appear in different studies ($p_{\text{overlap},t}=0$), $D_{t}=1.0$, preserving their full weight. For example, 12% of habit-related articles also reference other targets $\left(p_{\text{overlap},t}=0.9\right)$, then D=0.50+0.50(1-0.12)=0.55, meaning the adjusted prevalence of habit is reduced by 0.6106. $D_{t}$ is then used to adjust $P_{t}=0.1195$, yielding $P_{t}^{*}=0.0615$ after the normalization.

### 2.4 | Target Performance: Behavior Efficacy of Intervention Targets

Efficacy data were obtained from Albarracín et al. (2024), which synthesized odds ratios (*OR*s) for behavior change from meta-analyses of interventions across multiple domains (e.g., health and the environment). $E_{t}$ refers to these efficacy values.

Prevalence-weighted efficacy was computed as:

$$E_{t}^{\text{weighted}}=\left({OR}_{t}\right)\times P_{t}^{*}.$$

To facilitate interpretation across targets, these prevalence-weighted values were then normalized to a 0–1 range using min–max normalization:

$$E_{t}^{\prime }=\frac{E_{t}^{\text{weighted}}\;-\;{min}_{t}\left(E_{t}^{\text{weighted}}\right)}{{max}_{t}\left(E_{t}^{\text{weighted}}\right)\;-\;{min}_{t}\left(E_{t}^{\text{weighted}}\right)}.$$

This transformation preserves the relative differences in prevalence-weighted efficacy while eliminating distortions caused by extreme *OR* values and the asymmetric scaling of odds ratios (< 1 vs. > 1). Higher normalized 0–1 values thus indicate greater overall influence of a given target on behavioral change within the intervention literature (see Figure 1). Although we report efficacy in *OR* metric for descriptive purposes, all other indices are based on the normalized target efficacy, $E_{t}^{\prime }$.

### 2.5 | Target Efficiency: Cost-Effectiveness of Intervention Targets

Cost-effectiveness was quantified by incorporating empirical estimates of economic efficiency from Beard et al. (2022) with the prevalence of intervention targets. Cost-effectiveness estimates were available for all targets except trustworthiness. As a result, the trustworthiness target was retained for efficacy but excluded from cost calculations.

We first converted GBP to USD using 1.32 as the conversion rate. We then used USD–adjusted cost per quality-adjusted life year (QALY) values for each target. CE_*t*_ is the value for each target obtained from Beard et al. (2022). These values can be compared with the share of $50,000 QALY benchmark, a commonly used standard for health programs.

To obtain prevalence-weighted cost-effectiveness for each target, we then multiplied the raw cost-effectiveness values by the corrected (deflation–adjusted) prevalence of that target in the intervention literature:

$${CE}_{t}^{\text{weighted}}={CE}_{t}\times P_{t}^{*},$$

where ${CE}_{t}$ is the mean cost (or savings) in USD per QALY for the target t, which varies from 1 to 16, and $p_{t}^{*}$ is its prevalence after correcting for co-occurrence with other targets. This weighting ensured that targets that appear more frequently, and therefore exert more influence on intervention practice, contribute proportionally to the overall estimate. Negative values indicate cost savings, while positive values represent additional costs per QALY gained. ${CE}_{t}^{\text{weighted}}$ was used in the remainder of the paper.

### 2.6 | Disciplinary Centrality

We used GPT-5 to obtain ratings of the centrality of each of the 16 constructs for 14 different disciplines. The actual prompt was as follows:

Please rate how much each of the following disciplines contributed to defining, conceptualizing the operation, and measuring the following variables. The disciplines are social psychology, developmental psychology, clinical psychology, cognitive psychology, psychology, communication, sociology, economics, neuroscience, medicine, public health, biology, engineering, and chemistry. The variables are 16 intervention targets and sample terms using the keywords list in the attached table. Please use this scale: 1–5 scale (1 = *minimal/mostly outside the discipline’s core*; 2 = *peripheral/applied occasionally*; 3 = *moderate/regular but not defining*; 4 = *strong/frequent contribution*; and 5 = *central/foundational contribution*).

We thus obtained GPT ratings for each discipline for each target. For example, social support received a rating of 5 for psychology and sociology, but a rating of 1 for biology and chemistry. The list of target keywords provided to GPT appears in the Supporting Information S1.

### 2.7 | Disciplinary Weights

Each discipline’s total contribution to the behavioral intervention corpus was estimated by combining (a) the centrality of each target to that discipline and (b) the target’s efficacy or cost-effectiveness, $E_{t}^{\prime }$ and ${CE}_{t}$. First, the relative weight of discipline d for each target t was obtained as the rescaled centrality of that discipline in the disciplinary centrality ratings matrix:

$$W_{d,t}=\frac{r_{d,t}\;-\;1}{4},$$

where $r_{d,t}$ represents the 1–5 rating of how central the target t (1–16) is to the discipline d (1–14). This mapping sets 1 = 0 (“outside the discipline’s core”) and 5 = 1 (“foundational”), maintaining linear spacing among intermediate levels and truncating any minimal ratings to 0.

The total disciplinary weight across all targets was defined as

$$W_{d}^{\text{total}}=\sum_{t=1}^{T}W_{d,t},$$

where W is the weight of each target, d is the discipline, and t is each target out of the total of 16 targets.

### 2.8 | Discipline-Level Indices

#### Discipline-Level Efficacy.

Each discipline’s efficacy score reflects how strongly the discipline contributes to intervention efficacy in the literature, namely, the behavioral *performance* of the intervention targets central to a discipline. The discipline-level efficacy index was computed as each discipline’s normalized efficacy average:

$$E_{d}=\frac{\sum_{t}W_{dt}E_{t}^{\prime }}{\sum_{t}W_{dt}},$$

where $E_{t}^{\prime }$, the normalized efficacy of target t,W is the disciplinary weight for each target, and d is the discipline. This index, therefore, represents the expected empirical efficacy of an intervention whose components reflect the targets typically emphasized by that discipline.

We also included the discipline’s total share of the average efficacy of an intervention:

$$E_{d}^{\text{Share}}=\frac{\sum_{t}W_{dt}E_{t}^{\prime }}{\sum_{d}\sum_{t}W_{dt}E_{t}^{\prime }}.$$

#### Discipline-Level Cost-Effectiveness Index.

Discipline-level cost-effectiveness was computed analogously, using the discipline’s normalized weighted average:

$${CE}_{d}=\frac{\sum_{t}W_{dt}{CE}_{t}^{\text{weighted}}}{\sum_{t}W_{dt}},$$

where ${CE}_{t}^{\text{weighted}}$ is the prevalence-weighted cost-effectiveness of a target, W is the disciplinary weight of target t, and d is the discipline. This metric reflects the expected average *economic efficiency* of the targets most central to that discipline within the discipline, rather than the cost per individual study.

We then calculated the share:

$${CE}_{d}^{\text{Share}}=\frac{\sum_{t}w_{dt}{CE}_{t}^{\text{weighted}}}{\sum_{d}\sum_{t}w_{dt}{CE}_{t}^{\text{weighted}}}.$$

#### 2.8.1 | Summary of Weighting Chain

The full computation sequence from empirical to disciplinary-level efficacy and cost-effectiveness is as follows: target prevalence → target efficacy (or cost-effectiveness) → disciplinary centrality rating → disciplinary centrality weight → discipline-level contribution. Each intervention target thus progresses from bibliometric frequency to performance and efficiency metrics, to GPT-based centrality ratings, and, finally, to discipline-level integration (see Figure 1).

## 3 | Results

Our analyses first described the prevalence of intervention targets in the literature as well as the associations between intervention targets and disciplines. We then estimated the contribution of different disciplines by weighting the target efficacy and target cost-effectiveness in USD by the target’s disciplinary weights. The discipline-level values represent expected efficacy and cost-effectiveness values rather than precise monetary returns. All values are averages across *diverse* interventions in the published literature. Efficacy was estimated across all interventions summarized by Albarracín et al. (2024), which included workplace interventions, health interventions, and environmental behavior programs, among others. In contrast, cost-effectiveness was estimated based on *health* intervention data (Beard et al. 2022).

### 3.1 | Prevalence of Behavior Change Intervention Targets

**FIGURE 2 |:**
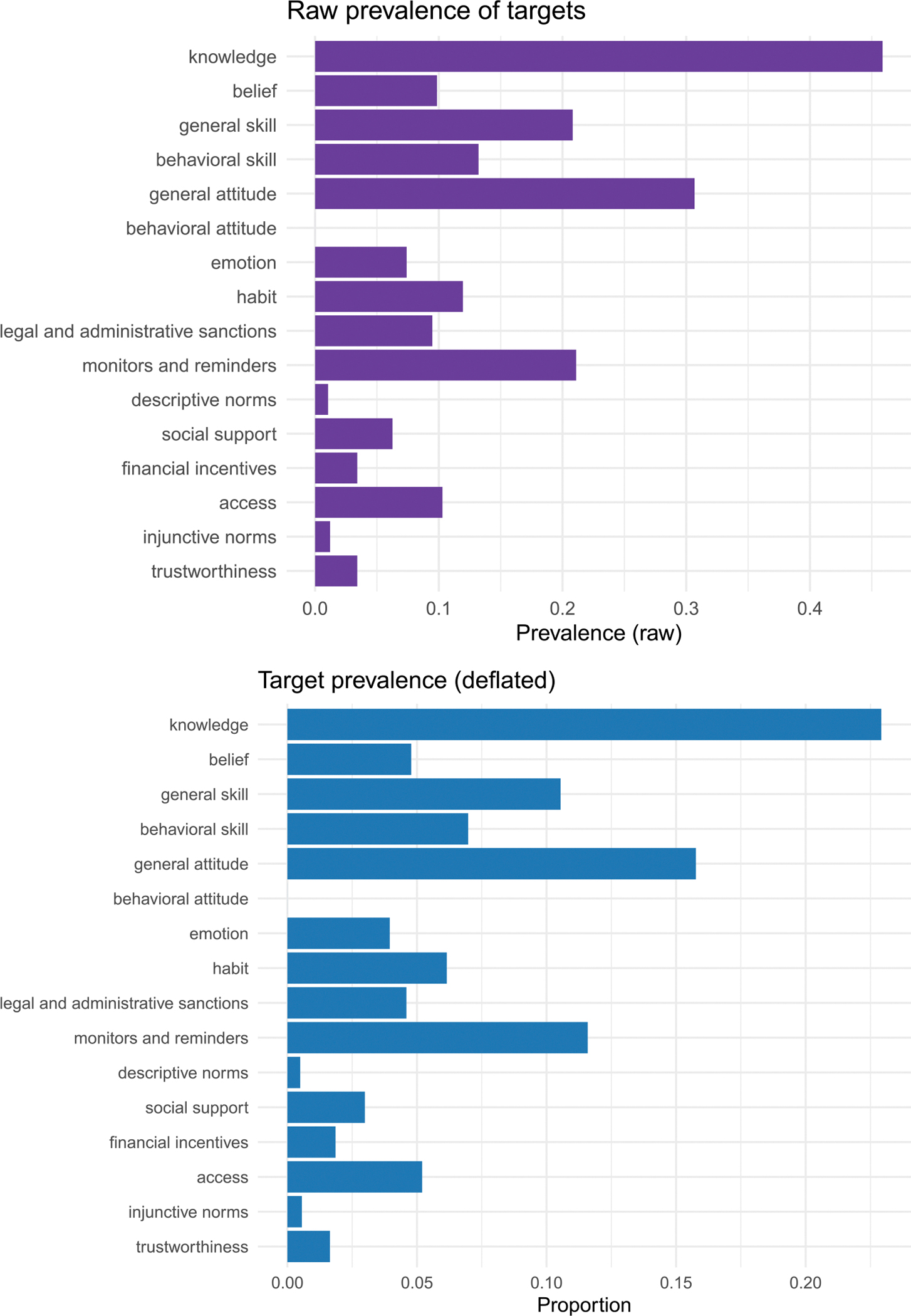
Raw ($P_{t}$; Top Panel) and Corrected (deflation–adjusted; $P_{t}^{*}$; Bottom Panel) prevalence of behavioral intervention targets across 4284 studies. Deflation adjusts for target co-occurrence in indexed abstracts and titles, providing a conservative estimate of target prominence.

The literature referencing experimental tests of behavior change interventions is extensive, comprising a total of 4284 papers. Figure 2 presents the raw and corrected (deflation-adjusted) prevalences of those articles that reference the targets in Table 1. As shown, these targets were referenced in numerous intervention articles, with each having an average prevalence of 12% in the literature. The interventions most often addressed knowledge, general attitudes, monitors and reminders, general skills, behavioral skills, social support, and access.

### 3.2 | Disciplinary Centrality to Intervention Targets

**FIGURE 3 |:**
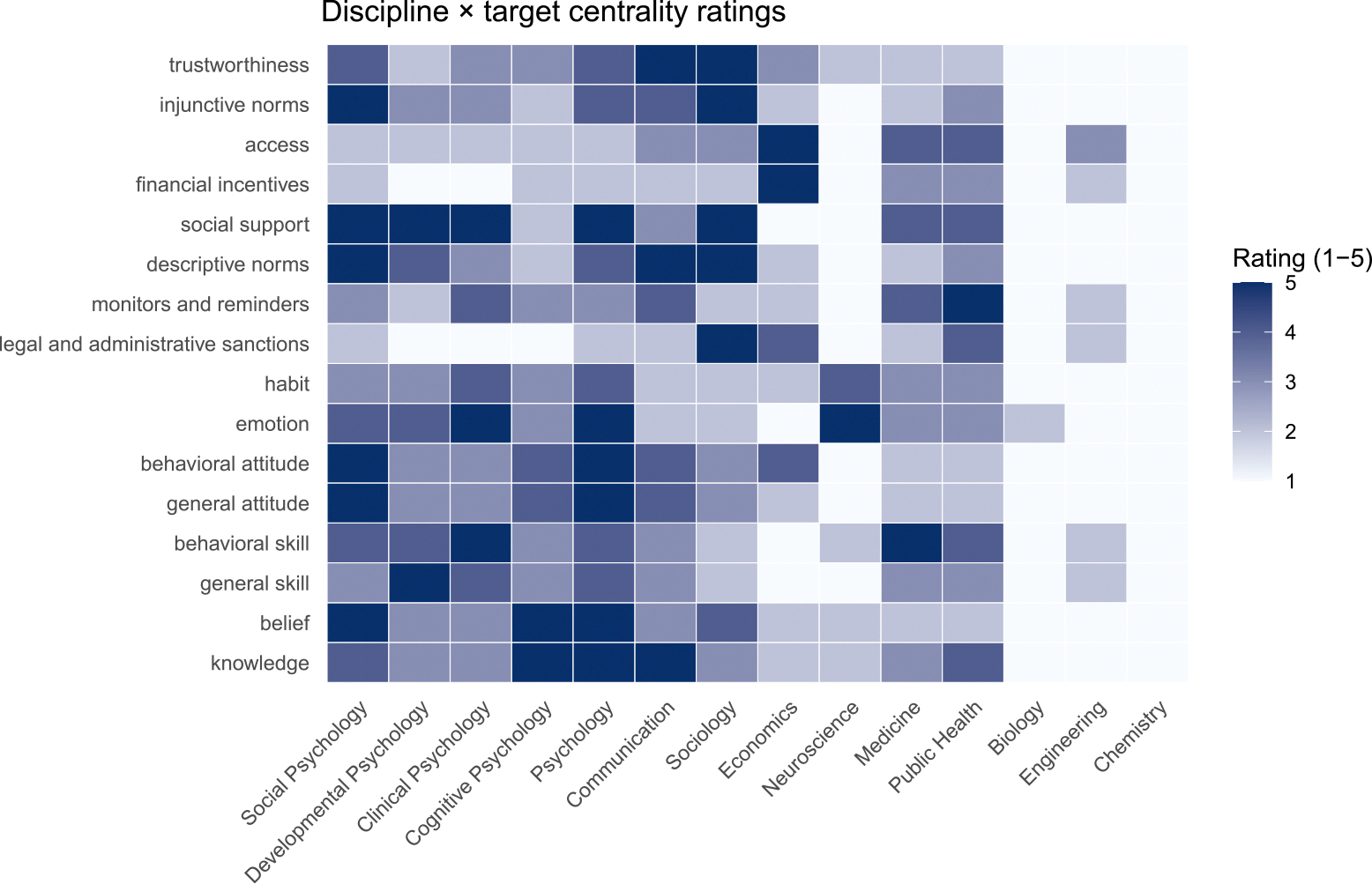
Associations between targets and disciplinary centrality ratings. GPT Ratings were provided on a 1 = 0 (“outside the discipline’s core”) and 5 = 1 (“foundational”) scale. Darker shades represent stronger disciplinary emphasis. These ratings were used in computing disciplinary weights and discipline-level efficacy and cost-effectiveness indices.

A heatmap representing the GPT-based ratings for the 14 disciplines is presented in Figure 3. As shown, within subdisciplines of psychology, social psychology played a central role (score of 5) in conceptualizing and measuring six targets: general attitudes, beliefs, behavioral attitudes, injunctive norms, descriptive norms, and social support. Psychology was central to knowledge, general attitudes, beliefs, emotions, behavioral attitudes, and social support. Sociology was central to legal and administrative sanctions, trustworthiness, injunctive norms, descriptive norms, and social support, while communication was central to knowledge, trustworthiness, and descriptive norms. The remaining seven disciplines played a central role for six targets: (a) economics for financial incentives and access, (b) neuroscience for emotions, (c) medicine for behavioral skills, and (d) public health for monitors and reminders. Finally, as one might expect, engineering, biology, and chemistry were not central to any of these intervention targets. Engineering had some association with access, whereas biology and chemistry were rated as playing no role.

### 3.3 | Intervention Efficacy

#### Overall and Target Efficacy.

**FIGURE 4 |:**
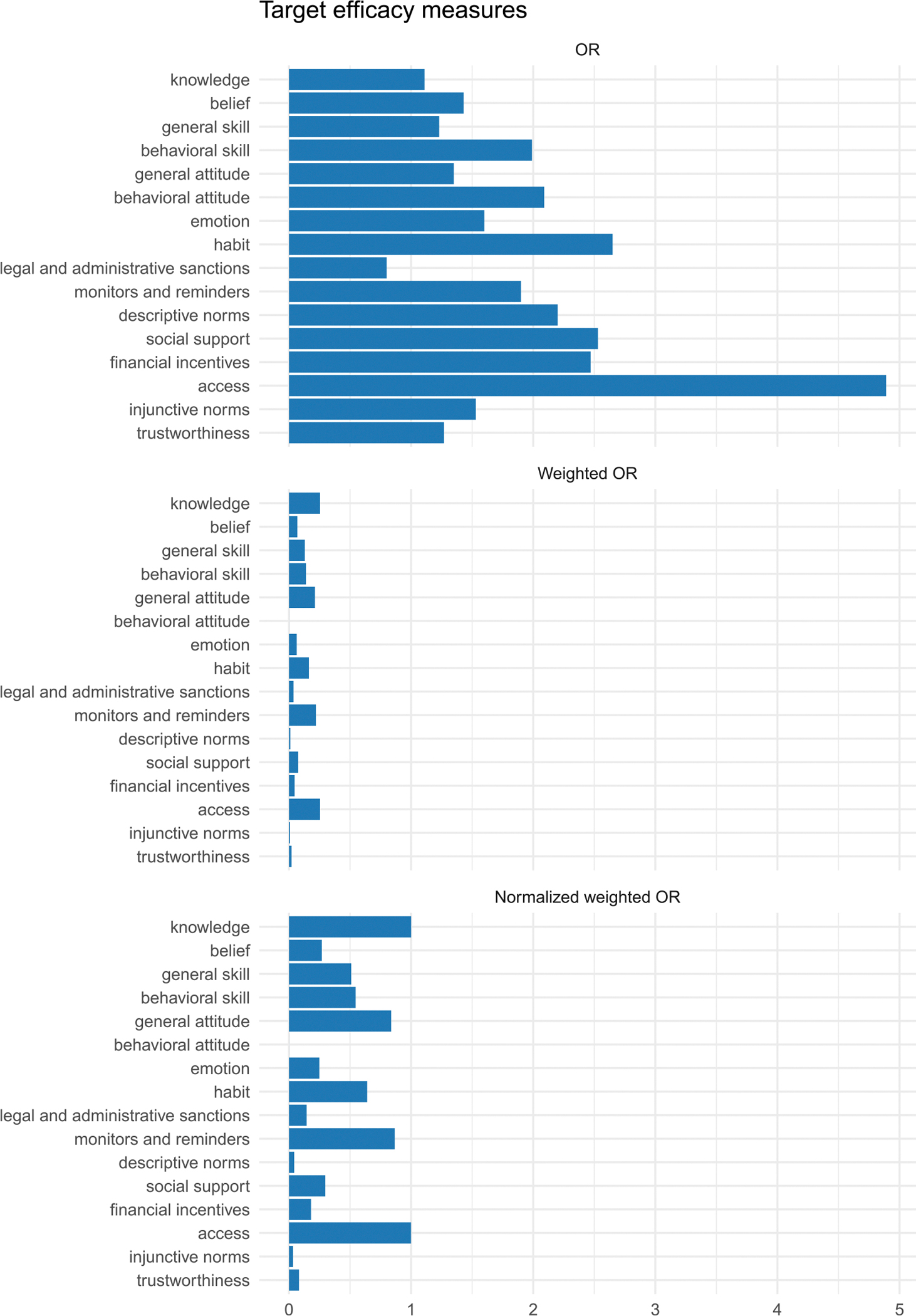
Target efficacy. Unweighted ORs ($E_{t}$; Top Panel), prevalence-weighted (OR) efficacy ($E_{t}$weighted; Middle Panel), and normalized prevalence-weighted efficacy ($E_{t}^{\prime }$; Bottom Panel). Higher values indicate a stronger performance in changing behavior.

The target efficacy data in our analyses (Albarracín et al. 2024) are summarized in the top panel of Figure 4. In terms of the raw *efficacy* (*E*_*t*_), targeting access, social support, and habits had the strongest impact, followed by interventions targeting emotional processes, behavioral skills, and behavioral attitudes. In contrast, interventions focusing on knowledge, beliefs, general attitudes, or general skills produced the least behavior change.

In our analyses, however, we not only examined raw efficacy but also weighted target performance by the prevalence of each target in the literature ($E_{t}$weighted). Considering the sum of the prevalence-weighted efficacy across targets, the intervention literature yields an OR of 1.70, which corresponds to a medium effect size, Cohen’s d=0.29. The collective disciplinary efforts that inspire behavior change intervention strategies are, therefore, of value in terms of total behavior change performance.

The prevalence-weighted efficacy per target ($E_{t}$ weighted and $E_{t}^{\prime }$) is also revealing (see second and third panels in Figure 4). As shown, different intervention targets make varying contributions, depending on their prevalence in the literature and their efficacy. Access and knowledge had the highest prevalence-weighted efficacy indices, followed by general attitudes, monitors and reminders, habits, and skills, with all other targets making smaller contributions to the collective performance of the behavioral interventions in the literature.

#### Discipline-Level Contribution.

**FIGURE 5 |:**
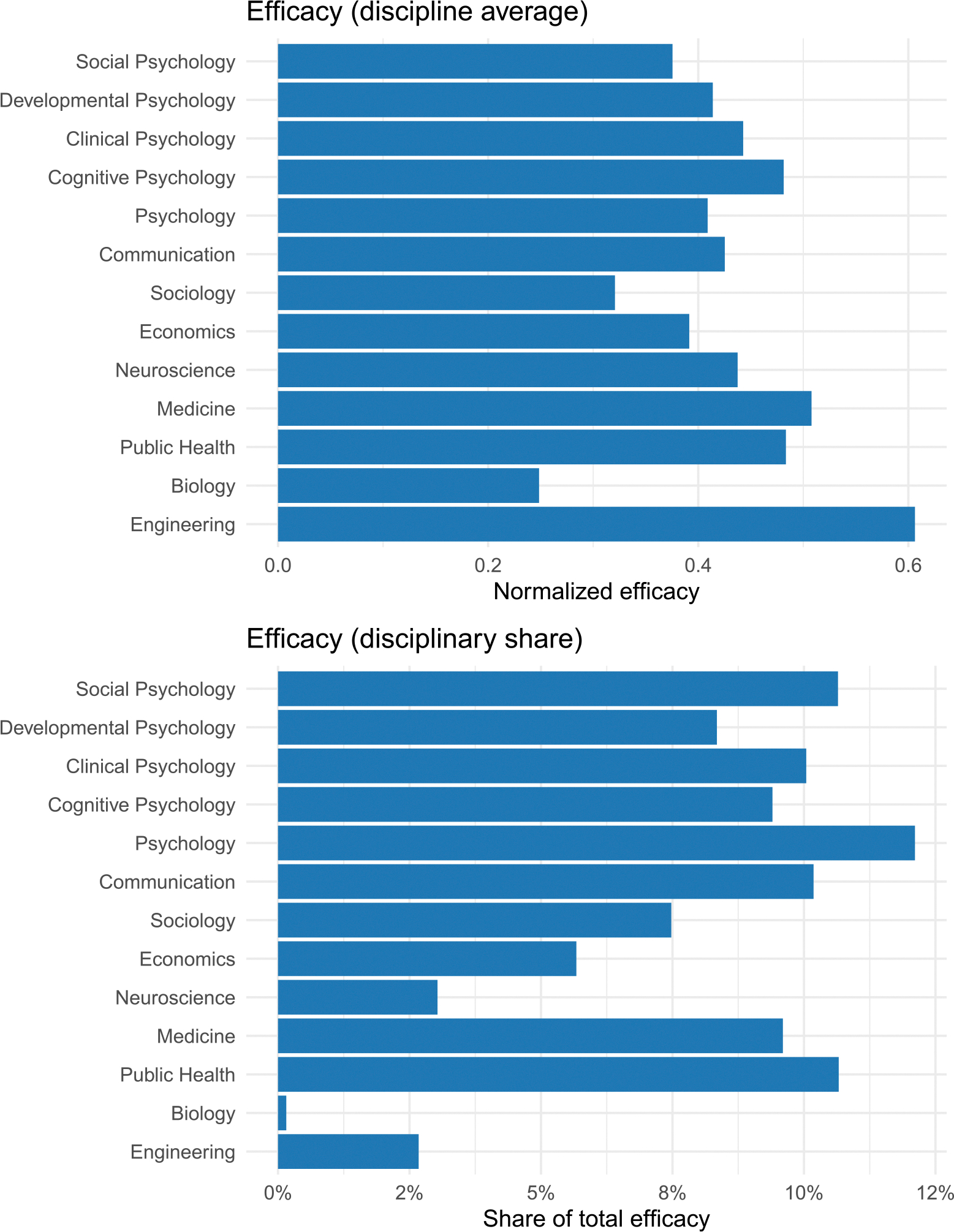
Disciplinary-level efficacy. Values reflect average (*E*_*d*_; top panel) and total (*E*_*d*_ Share.bottom panel) performance potential given each discipline’s emphasis.

The discipline-level efficacy indices ($E_{d}^{\prime }$) appear in Figure 5. As shown, psychology had the best performance in terms of share among all disciplines, followed by communication, public health, and medicine. Within psychology, social and cognitive psychology made sizable contributions.

### 3.4 | Intervention Cost-Effectiveness

**FIGURE 6 |:**
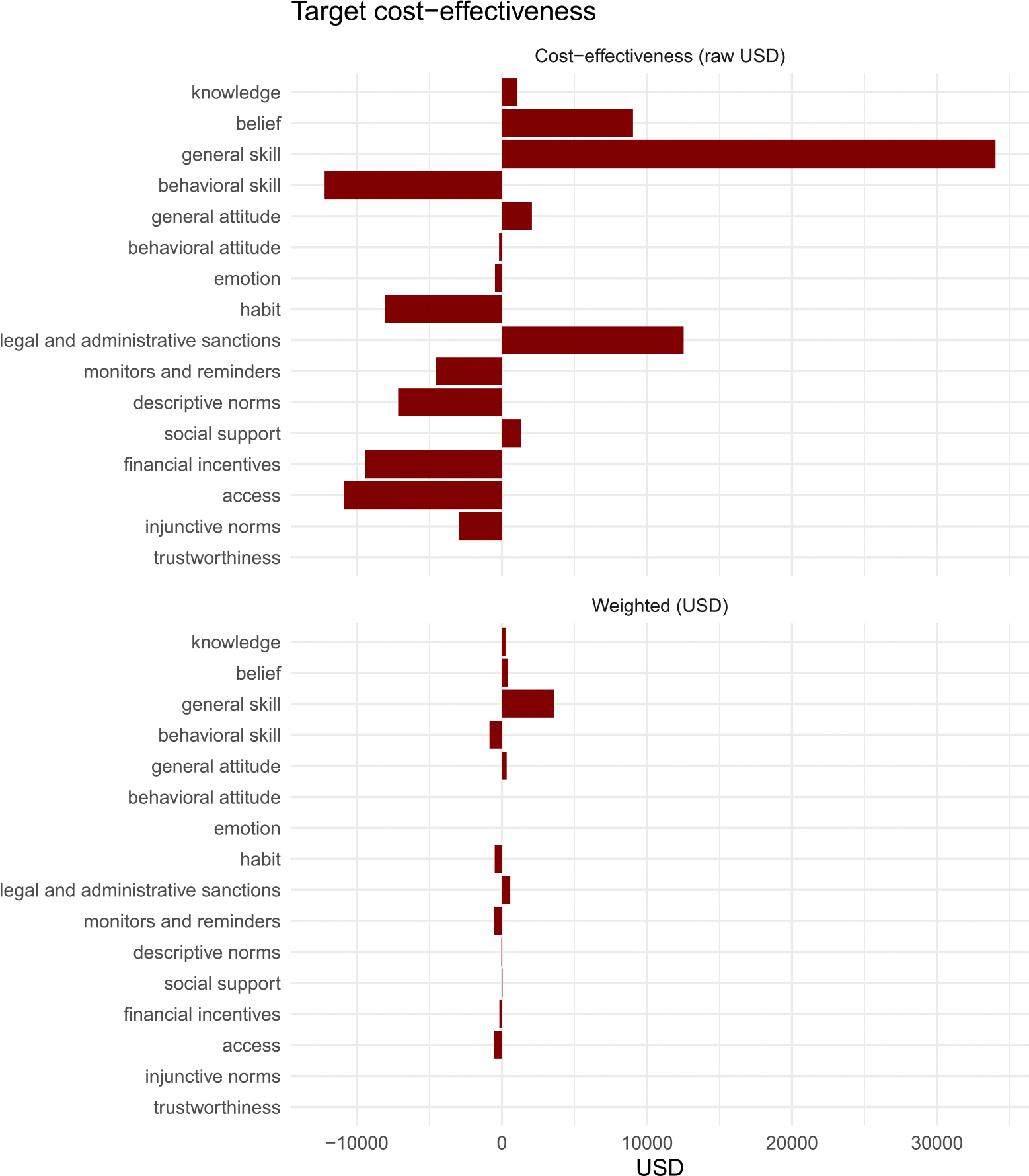
Target cost-effectiveness in USD. Lower values indicate greater efficiency (less cost per behavioral impact). The top panel shows average cost data (Beard et al. 2022; the bottom panel shows prevalence-weighted values (${\mathrm{CE}}_{t}$). Negative values imply savings, and lower values imply more cost-effective (efficient) targets.

**FIGURE 7 |:**
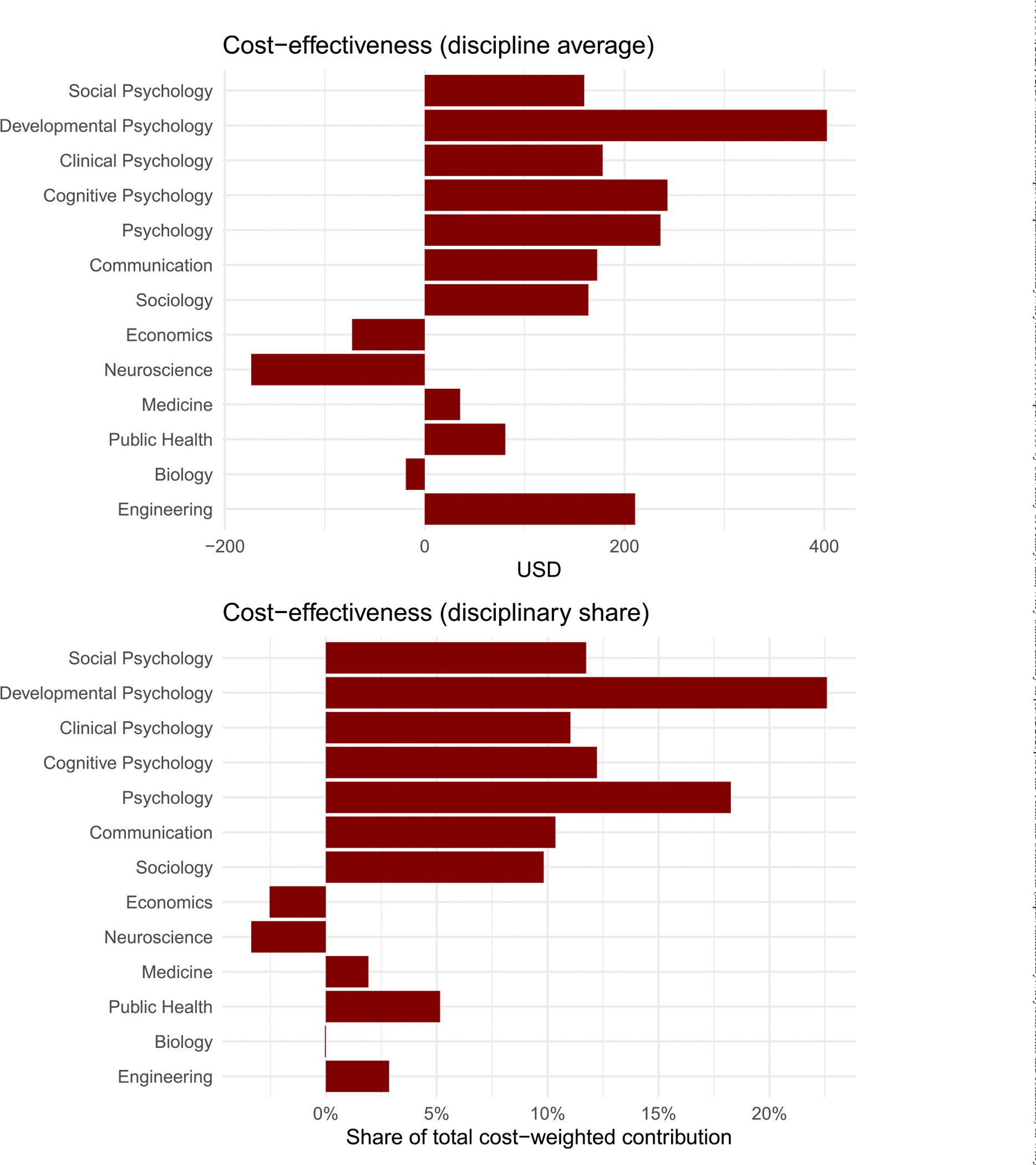
Discipline-level cost-effectiveness in USD (CE_*d*_). Lower values indicate greater economic efficiency. Bars represent the expected average costs (top panel) and share of total costs (CE_*d*_ Share; bottom panel) of interventions with targets typically emphasized by each discipline. Negative values imply savings, and lower values imply more cost-effective (efficient) disciplinary contributions.

The prevalence-weighted cost-effectiveness data separated by target and discipline appear in Figures 6 and 7.

#### Overall and Target Level.

When considering target cost-effectiveness by intervention target (${\mathrm{CE}}_{t}$ see Figure 6), the total QALY across all targets was $2517.41, implying a highly cost-effective average contribution under the $50,000 QALY threshold. Considering target differences, promoting access, habits, monitors and reminders, and behavioral skills yielded savings. Interventions designed to promote general skills are the most expensive. For example, a general skills health-behavior intervention costs $34,028, which remains below the $50,000 QALY threshold. The cost-effectiveness share of general skills interventions is $3587.10, representing an 11% corrected (deflation-adjusted, normalized) share of the literature, with a cut-off of $5500 (11% of the $50,000 QALY threshold for the total intervention). Thus, all intervention targets were efficient.

#### Discipline Level.

When considering discipline-level cost-effectiveness (CE_*d*_; see Figure 7), economics and neuroscience were associated with the most cost savings on average, as might be expected due to the emphasis on access and habits, respectively. However, as implied by a total average QALY of $1516.59 across all disciplines, the combined contributions were significantly below the $50,000 QALY threshold, indicating that they were efficient.

### 3.5 | Sensitivity Analyses

We conducted three types of sensitivity analyses. The first was to estimate cost-effectiveness after increasing the estimated QALY to 1 SD above the current figures. This analysis did not alter the conclusion that the target- and discipline-level indices demonstrated efficiency in relation to the $50,000 QALY benchmark. We also tested various GPT prompts and keywords, both for Web of Science searches and R searches, as well as GPT ratings. Additionally, we compared the experimental literature with the broader literature, inclusive of literature reviews. Our results were robust to these methodological variations.

## 4 | Conclusions and Recommendations

The quantitative evidence presented in this paper suggests that, overall, the intervention literature yields excellent performance in terms of behavioral change outcomes. The total of the prevalence-weighted OR efficacy index suggests that approximately 70% of interventions targeting the factors outlined in Table 1 outperform their control counterparts. This performance is grounded in scientific research on access, social support, descriptive norms, habits, as well as general attitudes and knowledge (see Figure 4), all areas where psychology plays a central role.

If we develop an intervention with the targets in Table 1, what would its expected cost-effectiveness be? Our results, as shown in Figure 7, indicate that emphasizing access, habit formation, and reminders yields the largest cost savings, whereas focusing on skill and general attitude change remains cost-effective but is less efficient in monetary terms. For these reasons, economics and neuroscience produce relatively more cost savings than other disciplines, although all sciences inform efficient behavioral change interventions.

### 4.1 | Caveats

We were interested in quantifying the influence of different disciplines on socially beneficial behaviors through their impact on behavior change intervention targets. One could achieve this objective by examining the outcomes from each trial. However, given thousands of studies, such an analysis would prove impractical. The approach taken here was to estimate the prevalence of studies testing specific intervention targets (e.g., injunctive norms) and then link it to the typical efficacy and cost-effectiveness of an intervention. This process, however, involves aggregating individuals within trials and trials within research syntheses (e.g., Albarracín et al. 2024). Therefore, given the well-known risks of the ecological fallacy, these aggregated data should be taken merely as an illustration of averages.

None of the contribution markers offered here is without limitations. To begin, bibliometric data are highly imperfect. They depend on full coverage of keywords and comprehensive indexing of publications, neither of which exists. Therefore, the total citations and any estimates derived from bibliometric data are provided for comparison purposes, rather than in absolute terms.

The ratings produced by GPT are known to have cultural biases. Therefore, the results from these ratings could be replicated with other centrality assessments provided by researchers, editors, and funders. Similarly, the data from research syntheses have publication bias and might have other distortions as well.

Although we present cost-effectiveness data, socially beneficial behaviors, including health behavior, go beyond financial costs (Coelho 2023). Therefore, intervention performance in terms of behavior change is more important to human lives than efficiency. Thus, efficacy should receive the utmost attention in determining what research to fund or what behavioral change programs to implement.

Within assessments of performance, behavioral intervention research is only part of a broader universe of social and behavioral impacts. First, our analysis only considers behavior change, even though psychology and other disciplines inform other programs, including mental health treatment. Second, we estimated the impact of behavior change interventions through intervention research. As a result, our approach is silent to the informal effects of psychology or other disciplines on the much larger body of behavioral interventions implemented in the community, outside of randomized controlled trials.

Another constraint stems from the efficacy and cost-effectiveness data themselves. The cost-effectiveness estimates used in this analysis were obtained by Beard et al. (2022). As a result, cost-effectiveness is limited to health interventions, and one intervention target (i.e., trustworthiness) lacks cost-effectiveness estimates. This introduces uncertainty about the cost-effectiveness estimates.

Finally, although Albarracín et al.’s (2024) review tried to identify efficacy estimates from isolated components, when possible, these figures are not always statistically independent. Therefore, both efficacy and effectiveness estimates should be used comparatively, also remembering that real interventions vary, whereas our results are based on averages.

### 4.2 | Federal Research Funding

**FIGURE 8 |:**
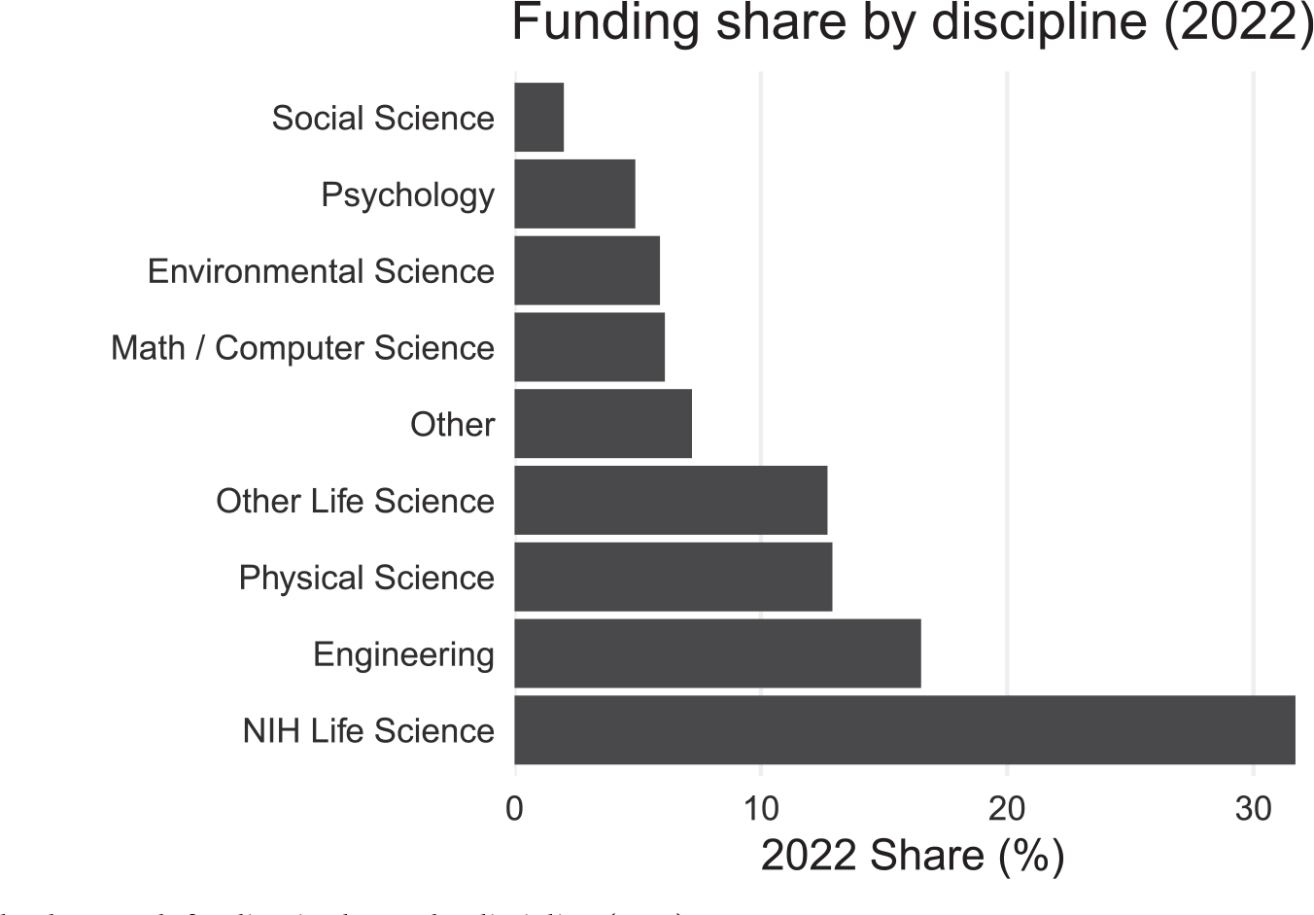
Federal research funding in the US by discipline (2022).

A final question concerns the level of federal Research and Development (R&D) funding for psychology in the United States. Considering the total federal R&D budget in recent years, which appears in Figure 8, the allocation to psychology has been between 3.1% and 4.9% (Stamm et al. 2025). These levels seem insufficient to meet the demand for innovation in the science of behavioral change, an area that remains a national priority (Tallie and Popkin 2025).

**FIGURE 9 |:**
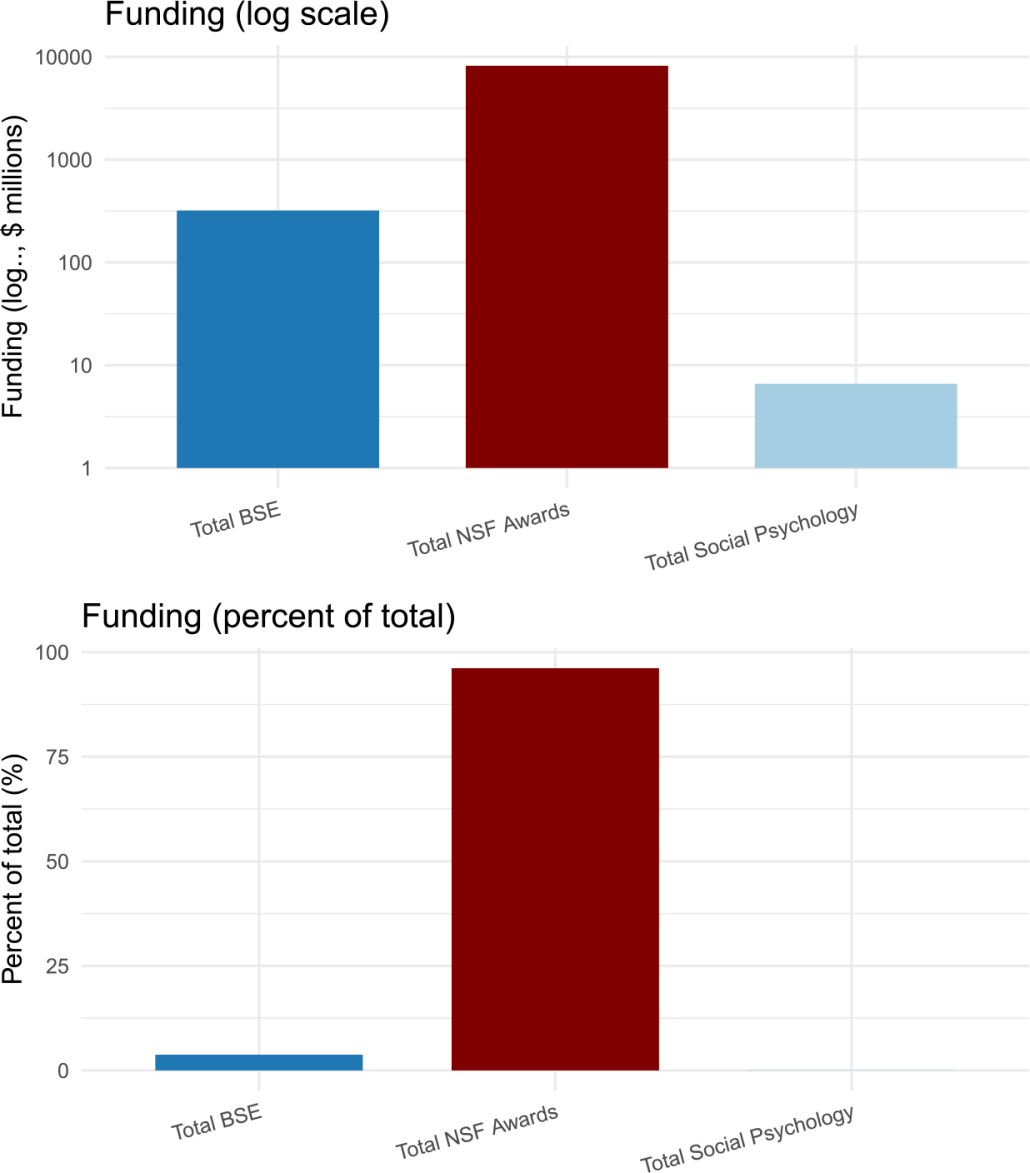
National science foundation awards for the behavioral, social, and economic sciences (SBE) and social psychology (2023).

Another question concerns allocations specific to social psychology. The data in Figure 8 encompass all areas of psychology, including not only social psychology but also developmental, clinical, and cognitive psychology, among others. A more precise indication of the federal investment in social psychology can be obtained by considering awards from the National Science Foundation (NSF), where a specialized social psychology program exists as part of the Social, Behavioral, and Economic (SBE) Sciences Division. The NSF awards to social psychology are presented in Figure 9, alongside the total and SBE awards, expressed as log-transformed millions of USD (top) and percentages (bottom) in 2023. Considering NSF’s total awards (~$8200 million) and the subtotal of SBE awards (~$320 million), the $6.6 M in social psychology awards represent only 0.1% of the total awards and only 2% of the SBE awards. In other words, despite its measurable influence, social psychology remains an underfunded field, receiving a trivial portion of the total federal research support in the United States. This discrepancy suggests a substantial missed opportunity for public benefit.

Increasing investment in psychological research would not only be equitable, given its demonstrated impact, but also economically sound. Psychology-inspired programs save lives, improve public health, and offer scalable, cost-effective solutions to persistent societal challenges. To realize this potential, the field must continue to produce theoretically informed, empirically grounded, and policy-relevant work, while funders and policy-makers must recognize and support its value. By aligning funding levels with demonstrable impact, the US could unlock new, scalable, evidence-based strategies to advance public welfare.

## Supplementary Material

supplementary material

Supporting Information

Additional supporting information can be found online in the Supporting Information section.

**Supporting Information S1:** spc370109-sup-0001-suppl-data.docx.

## Figures and Tables

**TABLE 1 |:** Behavior-change targets: Definitions and sample interventions (adapted from Albarracín et al. 2024).

Determinant	Definition	Representative example interventions

Individual change targets		
Knowledge	Collection of facts about an object or behavior, which can include information about the properties and consequences of a particular object or event, such as a virus or pollution. Knowledge links an object or behavior to an attribute or event with absolute certainty.	Health education
		Didactic instruction about climate change in schools
General skills	Cognitive or overt routines that allow individuals to carry out a variety of specific behaviors. They involve broad capacities such as controlling attention during tasks and being able to inhibit temptations when behaviors require high levels of self-control.	Behavior change programs emphasizing the need to train general skills that might help individuals control undesirable behaviors
General attitudes	Evaluations of objects, persons, and events. For example, prejudice is a negative judgment of a group as the attitude object, and an attitude toward cars is a positive or negative evaluation of cars as the attitude object. This type of attitude is often termed “attitude toward the target”.	Mass-media health-promotion campaigns about a behavior
		Interventions aimed at weakening associations by instilling goals and threat
Beliefs	Subjective assignments of probability that an object or behavior has a given attribute or outcome.	Messages that explicitly introduce expectations about a behavior
		Growth mindset interventions in academic settings
Emotions	Visceral feelings (e.g., happiness or fear) associated with a particular object, person or event. Experiencing fear of climate change or disgust about a particular group of individuals are examples of emotion.	Emotional appeals that sensitize audiences to risks and include discussion of the threat posed by a problem or the audience's susceptibility to it
Behavioral skills	Routines that enable people to execute a target behavior, often reflected in higher levels of perceived control or efficacy concerning the behavior.	Practicing and receiving feedback on the behavior and performing homework related to the behavior
		Asking individuals to formulate implementation intentions
Behavioral attitudes	Evaluations of a behavior as good or bad. For example, whereas an attitude toward cars is a general attitude, an attitude toward driving a car for transportation is a behavioral attitude. This type of attitude is often referred to attitude toward the behavior.	Mode of questioning designed to uncover and reduce attitudinal ambivalence toward a particular behavior
Habits	Behavioral routines that have acquired features of automaticity, meaning that they occur efficiently, without awareness, or continue even without intention and after they are no longer adaptive.	Training to stop a behavior when faced with temptations
		Introducing environmental regularity to promote habit formation
		Distracting oneself from behavioral cues
Social-structural change targets		
Legal and administrative sanctions	Legal and administrative instruments to prescribe, ban, or sanction a behavior.	Banning smoking in public establishments
		Mandating vaccination
		Mandating sick pay
		Taxing pollution
Trustworthiness	Justice or fairness within an organization or government entity, which leads constituents to follow recommendations.	Providing channels for Latinx voters to voice their concerns
		Community-oriented policing that fosters nonenforcement interactions
Injunctive norms	Perceptions of the degree to which others support a person's behavior.	Messages that communicate that others approve of condom use
		Posting signs stating that taking the stairs is a good way to get some exercise
Monitors and reminders	Physical or digital instrument to track behavioral performance and remind users of the need to execute a behavior.	Clinical reminder system for promoting preventive care
		Digital watches and phone apps that promote physical activity
Descriptive norms	Frequency of a behavior in a particular population.	Comparative feedback such as a chart tracking one's energy consumption in relation to one's neighbors
		Using role models to promote a target behavior Posting signs stating that most people used the stairs
Material incentives	Providing financial or non-financial rewards in exchange for a behavior.	Paying people US$24 to receive the COVID-19 vaccine
Social support	Informational, instrumental, or financial help to facilitate a particular behavior.	Leveraging family or ad hoc groups to assist individuals to meet their physical activity goals
		Groups of Latina mothers led by “promotoras” who support and accompany each other during health promoting activities
Access	Material or logistic resources to facilitate the performance of a behavior.	Reducing co-payments for medication
		Providing health insurance
		Providing basic income
