# Supplementary material for: Scientific Impact on Socially Beneficial Behaviors: Impact and Efficiency Evidence From Behavior Change Interventions
Source: Soc Personal Psychol Compass. Author manuscript; Available in PMC 2026 May 20. (PMC13186442; doi:10.1111/spc3.70109)
Supplement: supplementary material [file NIHMS2144728-supplement-supplementary_material.docx]

**Supplemental Materials**

**Search for Behavioral Intervention Experiments in Web of Science**

AB=(intervention* OR program* OR campaign* OR "behavioral intervention*" OR "behavior modification" OR "behavioral treatment" OR "persuasive communication" OR "health promotion" OR "behavioral intervention" OR "behavioral strategy" OR “training” OR “education”)

AND

AB=("behavior change" OR "behaviour change" OR "behavioral change" OR "behavioural change" OR "habit formation" OR "habit change" OR "behavior adoption" OR "behavior maintenance")

AND

(AB=(experiment* OR trial* OR "randomized controlled trial" OR “RCT”))

NOT

(AB=(animal* OR meta* OR review OR synthesis OR commentary OR letter OR editorial OR protocol OR "case report" OR "systematic review" OR "literature review" OR "scoping review")))

**Target Keywords**

# 1) Knowledge

pat_knowledge <- c(

"\\bknowledge\\b",

"informational content",

"knowledge acquisition",

"informational exposure",

"informational intervention"

)

# 2) Belief

pat_belief <- c(

"\\bbelief\\b", "\\bbeliefs\\b",

"expectancy", "expectation",

"cognitive representation",

"mental model",

"\\bschema\\b",

"implicit theory",

"\\bworldview\\b"

)

# 3) general skill (include general skilly words, exclude behavioral-skill words)

pat_general_skill_include <- c(

"\\bskill\\b", "\\bskills\\b",

"\\bability\\b",

"\\bcompetence\\b",

"\\bcapability\\b",

"\\bpractice\\b",

"\\bmastery\\b",

"\\bproficiency\\b"

)

pat_behavioral_like <- c( # will use to exclude from general skill

"behavioral skill", "behavioural skill",

"performance skill",

"self-regulation skill",

"coping skill",

"communication skill",

"self[- ]efficacy",

"perceived self[- ]efficacy",

"perceived control",

"perceived competence",

"confidence in ability",

"behavioral capability",

"behavioural capability",

"self-confidence",

"action control"

)

# 4) behavioral skill (the complementary set)

pat_behavioral_skill <- c(

"behavioral skill", "behavioural skill",

"performance skill",

"self-regulation skill",

"coping skill",

"communication skill",

"self[- ]efficacy",

"perceived self[- ]efficacy",

"perceived control",

"perceived competence",

"confidence in ability",

"behavioral capability",

"behavioural capability",

"self-confidence",

"action control"

)

# 5) Attitude (general)

pat_attitude <- c(

"attitude", "attitudes",

"evaluation",

"evaluative belief",

"opinion", "opinions",

"preference", "preferences",

"valence",

"favorability",

"evaluative response"

)

**GPT Prompt for Disciplinary Centrality Ratings**

‘Please rate how much each of the following disciplines contributed to defining, conceptualizing the operation, and measuring the following variables. The disciplines are social psychology, developmental psychology, clinical psychology, cognitive psychology, psychology, communication, sociology, economics, neuroscience, medicine, public health, biology, engineering, and chemistry. The variables are 16 intervention targets and sample terms using the keywords list in the attached table. Please use this scale: 1-5 scale (**1** = *minimal / mostly outside the discipline’s core*; **2** = *peripheral / applied occasionally*; **3** = *moderate / regular but not defining*; **4** = *strong / frequent contribution*;  **and 5** = *central / foundational contribution*). Please provide a CSV file.’

| **Target** | **Example Keywords / Wildcards** |
| --- | --- |
| **Knowledge / Information** | knowledge, information, awareness, understanding, education, informational content, knowledge acquisition, informational exposure, informational intervention, health literacy, awareness of |
| **Belief** | belief, beliefs, health belief, changing beliefs, misconceptions, perceived risk, perceived severity, perceived susceptibility |
| **Attitude toward Object** | attitude toward, attitudes toward, attitude towards, attitudes towards, evaluation, opinion |
| **Attitude toward Behavior** | attitude to perform, attitude to engage, attitude to adopt, attitude toward the behavior, favorable attitude to, positive attitude to exercise, positive attitude to vaccination |
| **General Skill** | general skill, skills training, skill building, competence, capacity building, self-management skill |
| **Behavioral Skill** | behavioral skill, behavioural skill, practice the behavior, skill rehearsal, behavioral rehearsal, role play, problem solving skills |
| **Emotion / Mood** | emotion, emotions, emotional, affect, positive affect, negative affect, mood, anxiety, fear appeal, depression, emotional response |
| **Habit / Routine** | habit, habits, habitual, automaticity, routine behavior, breaking routines, implementation intention |
| **Trust / Justice / Ethics (Trustworthiness)** | trust, trustworthy, justice, ethics, credibility, truthfulness, trust in source, source credibility, trusted communicator, ethical, perceived credibility |
| **Law / Regulation / Deterrence** | law, laws, regulation, regulations, policy intervention, mandatory, legislation, government regulation, compliance, enforcement |
| **Monitor / Reminder** | reminder, reminders, prompt, cue, cues to action, monitoring, self-monitoring, feedback, text message reminder, electronic monitoring |
| **Injunctive Norms** | injunctive norm, social approval, moral norm, moral obligation, approval of others, people think I should, others expect, ought to, should do, perceived expectations |
| **Descriptive Norms** | descriptive norm, most people, other people do, people like me do, friends do, what others do, behavioral norm, prevalence of behavior |
| **Social Support** | social support, peer support, family support, community support, spousal support, support from friends, emotional support, instrumental support |
| **Incentive** | incentive, incentives, financial incentive, monetary incentive, cash incentive, voucher, conditional cash transfer, reward, payment for performance, lottery incentive |
| **Access / Default / Availability** | access, accessibility, availability, access to care, service availability, barrier to care, transportation barrier, default option, easy access |
